# Effect of *Cistanches Herba* Aqueous Extract on Bone Loss in Ovariectomized Rat

**DOI:** 10.3390/ijms12085060

**Published:** 2011-08-08

**Authors:** Haidong Liang, Fang Yu, Zhihong Tong, Zaiguo Huang

**Affiliations:** 1 Hands and feet microsurgery, Dalian Municipal Central Hospital, Dalian 116033, China; E-Mail: tongzhih37@sina.cn; 2 School of Medicine, Dalian University, Dalian 116033, China; E-Mail: yufangk7@sina.cn; 3 Orthopedics Department, Dalian Municipal Central Hospital, Dalian 116033, China; E-Mail: huangzg41@sina.cn

**Keywords:** ovariectomy, rat, bone, antioxidant, *Cistanches Herba*

## Abstract

To assess the ability of traditional Chinese medicine *Cistanches Herba* extract (CHE) to prevent bone loss in the ovariectomized (OVX) rat, *Cistanches Herba* extract (CHE) was administered intragastrically to the rats. Female rats were anesthetized with pentobarbital sodium (40 mg kg^−1^, i.p.), and their ovaries were removed bilaterally. The rats in the sham-operated group were anesthetized, laparotomized, and sutured without removing their ovaries. After 1 week of recovery from surgery, the OVX rats were randomly divided into three groups and orally treated with H_2_O (OVX group) or CHE (100 or 200 mg kg^−1^ daily) for 3 months. The sham-operated group (*n* = 8) was orally treated with H_2_O. After 3 months, the total body bone mineral density (BMD), bone mineral content (BMC), Bone biomechanical index, blood mineral levels and blood antioxidant enzymes activities were examined in sham-operated, ovariectomized and *Cistanches Herba* extract treated rats. Results showed that *Cistanches Herba* extract treatment significantly dose-dependently enhanced bone mineral density (BMD), bone mineral content (BMC), maximum load, displacement at maximum load, stress at maximum load, load at auto break, displacement at auto break, and stress at auto break, and blood antioxidant enzymes activities, decreased blood Ca, Zn and Cu levels compared to the OVX group. This experiment demonstrates that the administration of *Cistanches Herba* extract to ovariectomized rats reverses bone loss and prevents osteoporosis.

## 1. Introduction

Osteoporosis is a disease of aging associated with bone loss that often occurs without symptoms until microarchitectural deterioration becomes so significant that bone fracture occurs invariably at the point where a reduction in the bone mass is below what is required for bone integrity. Osteoporosis is of great importance in health care. Although reduced in mass, the bones are normal with respect to mineralization but histologically, there is a decrease in the thickness of the cortex and the number and size of tubeculae of the coarse cancellous bone [1–3].

Plenty of studies into this disease have been carried out in recent years. Accumulating data have indicated that the loss of estrogen at menopause is a major contributor to pathogenesis of the disease because this hormone is a principal negative regulator of osteoclast activity [4–6], and osteoclasts are the main cells responsible for bone remodeling in osteoporosis [7].

Postmenopausal osteoporosis is the most frequent metabolic bone disease, it is characterized by a rapid loss of mineralized bone tissue, disruption of trabecular architecture of the bone and changes in the crystalline properties of mineral deposits, which result in the structural failure (fracture) of sites rich in cancellous bone, such as the vertebrae, hip and distal forearm [8–11]. Estrogen deficiency is considered as the major determinant of bone loss in postmenopausal women. Ovariectomized rats and dogs have been used extensively in osteoporosis models. In 1996, the effects of incadronate on ovariectomized dogs [12] were reported.

*Cistanches Herba* (CH), holoparasitic plant of *Cistanche deserticola* Y.C. Ma, is classified as a tonifying agent in oriental traditional medicine and is commonly used to treat forgetfulness, loss of hearing, infertility, and chronic constipation. CH has various pharmacological activities, including antinociceptive, anti-inflammatory, and immuno-enhancing effects [13,14]. A previous study has reported that *Cistanches Herba* can guide bone mesenchymal stem cells (BMSCs) to differentiate into osteoblasts, which promises a favorable prospect for the treatment of osteoporosis and bone fracture disunion [15]. Chen *et al*. [16] reported that *Cistanches Herba* polysaccharides may promote the bone marrow cell cycle transition and the recovery of hematopoietic function in bone marrow depressed anemic mice, accelerate hematogenesis in rubrum strain and macronucleus strain.

In this study, bone mineral density (BMD), bone mineral content (BMC), Bone biomechanical index, blood mineral levels and blood antioxidant enzymes activities were examined in sham-operated, ovariectomized and *Cistanches Herba* treated rats to study the effects of treatment with *Cistanches Herba* extract on bone quality.

## 2. Materials and Methods

### 2.1. Preparation of Cistanches Herba Aqueous Extracts

*Cistanches Herba* was collected from the desert in alxa league of the Nei Monggol (Inner Mongolia) Autonomous Region in 2010.

The dried stalk of *Cistanches Herba* was extracted with distilled water at 100 °C for 2 h. The solution was centrifuged at 4000 r/min for 10 min. Residues were removed by filtration and the combined filtrate was evaporated by heating to obtain a desired concentration (2.1 g dry plant equivalent extract/mL). The yield of extraction was 13.7% (w/w). The aqueous extract was stored at 2–8 °C.

### 2.2. Animals

Thirty-two female Wistar rats of 3 months old, weighing between 240 and 270 g at the beginning of the experiment were used for the study. They were maintained under standard laboratory conditions with 12 h light and dark cycle, with free access to standard laboratory rat food and tap water.

### 2.3. Animal Experiments

Female rats were anesthetized with pentobarbital sodium (40 mg kg^−1^, i.p.), and their ovaries were removed bilaterally. The rats in the sham-operated group were anesthetized, laparotomized, and sutured without removing their ovaries. After 1 week of recovery from surgery, the OVX rats were randomly divided into three groups (*n* = 8 in each group) and intragastrically treated with H_2_O (OVX group), *Cistanches Herba* extract (CHE) (100 or 200 mg kg^−1^ daily) for 3 months. The sham-operated group (*n* = 8) was orally treated with H_2_O.

On the last day of the study, the animals were weighed and sacrificed, and blood samples were collected simultaneously. The femurs were dissected and divested of soft tissue for the measurement of biomechanical testing.

All experiments were carried out in accordance with research protocols established by the animal care committee of China.

### 2.4. Bone Densitometry

Femoral bone mineral content (BMC) and bone mineral density (BMD) were determined using a Hologic QDR-1000W DXA. The instrument was adapted for an ultrahigh-resolution mode with line spacing of 0.0254 cm, resolution of 0.0127 cm, and collimator of 0.9 cm diameter. The bones were placed in a petri dish. To simulate soft-tissue density surrounding the bones, tap water was poured around the bones to achieve a depth of 1 cm. Results are given for BMC and for the area measured. BMD of this area is calculated as BMC divided by area. In addition to results for total femur, the distal and midregion of the femur were analyzed as subregions [17].

### 2.5. Bone Biomechanical Testing

Femur bones were kept at 4 °C until determination of breaking strength using a 5-kN Flexure Fixture, configured for three-point-bending tests and attached to an Instron Universal Testing Machine Model 4502 equipped with a 10-kN load cell (Instron, Canton, MA, USA), as previously described [18]. The crosshead speed was 50 mm/min, and the data sampling rate was 10 samples/s. Maximum load, stress at maximum load, load at failure and stress at failure were determined in femurs using Series IX, v 8.08.00 software (Instron).

### 2.6. Biochemical Analysis

Blood Ca, Zn and Cu levels were measured by atomic absorption spectrophotometry.

Based on the method of King and Armstrong [19], alkaline phosphatase (ALP) activity was assayed using disodium phenyl phosphate as substrate. The color developed was read at 510 nm in a uv-vis spectrophotometer after 10 min. Activities of ALP was expressed as IU/L.

The blood malondialchehyche (MDA) concentration was determined using the method described by Jain *et al*. [20], based on TBA reactivity. Briefly, 0.2 mL erythrocyte pellets or supernatant obtained from tissues, 0.8 mL phosphate buffer (pH 7.4), 0.025 mL BHT and 0.5 mL 30% TCA were added to the tubes and mixed. After 2 h incubation at −20 °C, the mixture was centrifuged (4000× g) for 15 min. After this, 1 mL supernatant was taken and added to each tube, and then 0.075 mL of 0.1 M EDTA and 0.25 mL of 1% TBA were added. These tubes with Teflon-lined screw caps were incubated at 90 °C in a water bath for 15 min and cooled to room temperature. The optical density was measured at 532 for blood MDA concentration.

Activity of glutathione peroxidase (GSH-Px) was determined according to the method of Lawrence and Burk [21]. The assay mixture consisted of 2.0 mL of 75 mM phosphate buffer (pH 7.0), 50 μL of 60 mM glutathione, 0.1 mL of 30 units/mL glutathione reductase, 0.1 mL of 15 mM EDTA, 0.1 mL of 3 mM NADPH and the appropriate amount of tissue supernatant to a final volume of 3.0 mL. The reaction was started by the addition of 0.1 mL of 7.5 mM H_2_O_2_. The rate of change of absorbance during the conversion of NADPH to NADP^+^ was recorded spectrophotometrically at 340 nm for 3 min. GSH-Px activity for tissues was expressed as μmoles of NADPH oxidized to NADP^+^ min^−1^ mg^−1^ protein.

Activity of superoxide dismulase (SOD) was determined in the blood according to the method of Sun *et al*. [22]. The principle of the method based on the inhibition of nitroblue tetrazolium (NBT) reduction by using the xanthine-xanthine oxidase system as a superoxide generator. Activity was assessed in the supernatants of 10000 g of ethanol/chloroform (5/3, v/v) extracts of tissue homogenates (10%). One unit of SOD was defined as the enzyme amount causing 50% inhibition in NBT reduction rate.

The catalase (CAT) activity was measured using Abei’s [23] method. Serum was added to a cuvette containing 2.89 mL of a 50 mmol/L phosphate buffer (pH 7.0). The reaction was initiated by adding 0.1 mL of freshly prepared 30 mmol/L H_2_O_2_ to make a final volume of 3.0 mL at 25 °C. The decomposition rate of H_2_O_2_ was measured at 240 nm for 5 min on a spectrophotometer. One unit (U) of CAT was defined as the amount of enzyme required to decompose 1 nmol of H_2_O_2_ per min, at 25 °C and pH 7.8.

### 2.7. Statistical Analysis

The experimental results were expressed as the Mean ± SD for eight animals in each group. The biochemical parameters were analyzed statistically using one-way analysis of variance ANOVA, followed by Dunnett’s multiple comparison test (DMCT). *P* value of <0.05 was considered as statistically significant.

## 3. Results

Two animals expired during the course of the study. Consequently, numbers for the groups at study conclusion were: 8 in the sham control; 7 in the OVX group; 7 in the CHE-treatment group (100 mg kg^−1^ daily) and 8 in the CHE-treatment group (200 mg kg^−1^ daily). Two days after operation, rats’ food and water intakes were slightly lower than those before operation. No animal suffered from infection and anabrosis. No clinically evident adverse effects were observed in rats.

Before operation, there was no significant (*p* > 0.01) difference in rats’ body weight between groups. Body weight of the OVX group was significantly (*p* < 0.01) lower than that of the sham group (Table 1). *Cistanches Herba* extract administration at a dose of 100 and 200 mg/kg daily significantly (*p* < 0.05) increased rats’ body weight in the CHE-treated group (100 and 200 mg/kg daily). The data obtained from animals showed that there were statistically significant differences between the BMD and BMC of the OVX group and of the sham control (*p* < 0.01). As was expected, *Cistanches Herba* extract administration at a dose of 200 mg/kg led to a significant increase in the BMD and BMC of the CHE-treated group (*p* < 0.05; *p* < 0.01).

With three months of estrogen deficiency, maximum load, displacement at maximum load, stress at maximum load, load at auto break, displacement at auto break, and stress at auto break showed a tendency to decrease. Moreover, three biomechanical parameters all reach statistical significance (*p* < 0.01) compared to the sham group. *Cistanches Herba* extract treatment significantly dose-dependently increased maximum load, displacement at maximum load, stress at maximum load, load at auto break, displacement at auto break, and stress at auto break compared to the OVX group (Table 2).

The present data (Table 3) indicated that blood Ca, Zn and Cu levels in the OVX group were significantly (*p* < 0.01) increased compared to the sham control. Table 3 shows that only treatment with the CHE 200 dose had a significant (*p* < 0.05; *p* < 0.01) decreasing effect on all 3 parameters. The CHE 100 dose had a significant (*p* < 0.05) decrease only on Zn. The presence of *Cistanches Herba* extract normalized the levels of blood Ca, Zn and Cu nearly to the normal values of control.

Blood ALP activity, MDA contents significantly increased, and serum SOD, CAT and GSH-Px activities significantly decreased after the ovariectomy operation. The ALP activity and MDA levels, and SOD, CAT and GSH-Px activities obviously changed after treatment with *Cistanches Herba* extract treatment. The extracts showed a dose-dependent effect. The values of ALP and CAT activities were close to normal (Table 4, Table 5).

## 4. Discussion

Osteoporosis is one of the most common disorders associated with estrogen deficiency and aging. Menopause results in elevated bone turnover, an imbalance between bone formation and bone resorption, and net bone loss. Bone loss caused by estrogen deficiency is primarily due to an increase in osteoclastic bone resorption in both humans and experimental animals [24].

Although osteoporosis affects both older men and women, postmenopausal women have been the primary focus of osteoporosis [25,26]. A dramatic deficiency of female hormone is the most important factor to postmenopausal women osteoporosis. The OVX rat model is most commonly used in research on postmenopausal osteoporosis [27,28], in which ovariectomy results in an excess of bone resorption over bone formation initially and causes bone loss. OVX operation causes a significant body weight increase which could be a guidepost to evaluate the success of the operation [29]. At the end of the present study, the body weights of the OVX group were markedly reduced compared to those of sham group, and treatment with *Cistanches Herba* extract fully prevented these decreases. We suppose that OVX caused severe physiological discomfortt which affected the rats’ food intake. Subsequently, the body weight of the OVX group was markedly reduced.

Bone mineral density has been described as merely a surrogate measure of bone strength [30]; however, microarchitectural properties are a newly emerged marker for the evaluation of the true impact of a treatment on the quality of trabecular bone. Although low bone mass is a major risk factor for fracture [31], the preservation of trabecular bone architecture significantly contributes to bone strength and may reduce fracture risk beyond BMD and BMC, as demonstrated by a number of studies that have reported close correlations between microstructural properties and the biomechanical strength of bones [32,33]. In this work, BMD and BMC were found to be significantly different between the sham and ovariectomized groups. Treatment with *Cistanches Herba* extract significantly enhanced BMD and BMC in OVX rats. In addition, administration of *Cistanches Herba* extract still significantly dose-dependently increased maximum load, displacement at maximum load, stress at maximum load, load at auto break, displacement at auto break, and stress at auto break in CHE-treated groups compared to OVX group. These results indicated that femur bones from rats fed *Cistanches Herba* extract had higher load at failure, and thereby could be more resistant to stress fractures. In addition, rats in the OVX group had higher blood Ca, Zn and Cu levels than those in the sham group. Administration of *Cistanches Herba* extract had reduced blood Ca, Zn and Cu levels in OVX rats.

The notion that oxidative status is important in osteoporosis is consistent with the strong epidemiological association between osteoporosis and atherosclerosis, a disease in which oxidant stress plays a major role. Moreover, it was recently observed that there is an association in women between oxidant stress (measured by a biomarker of oxidative stress, 8-iso-PGF_2_-α) [34], antioxidant levels [35], and bone mineral density. Clinical studies that have examined the relationship between dietary and/or supplemental ascorbate and bone mass in humans also suggest an association with increased bone mass [36].

Since MDA has high reactivity towards amino groups, it inhibits the synthesis of nucleic acids and proteins and also deactivates the enzymes [37,38]. Thus, the decrease in femur antioxidant enzymes observed after OVX may is due to heightened lipid peroxidation. Antioxidant enzymes are involved in the defense system against free radical mediated tissue or cellular damage. They metabolize either free radicals or reactive oxygen intermediates to nonradical products. These enzymes include a family of glutathione dependent enzymes [39]. In this study, we report a significant decrease in the levels of antioxidant enzymes *viz*. SOD, GSH-Px, CAT in the blood of ovariectomized rats. These findings suggest that deficiency in ovarian hormones correlates with failure to combat oxidative stress. Administration of *Cistanches Herba* extract significantly dose-dependently enhanced blood SOD, GSH-Px, and CAT activities in OVX rats. These results appear to indicate a better protective effect of *Cistanches Herba* extract against oxidative stress induced during OVX.

In summary, *Cistanches Herba* extract is effective in preventing bone loss caused by ovarian hormone deficiency as seen from femur BMD, BMC values, and blood Ca, Zn and Cu levels. As osteoporosis is associated with oxidative stress, studies of this kind attest the importance of the therapeutic values of antioxidants. We suppose that *Cistanches Herba* extract improves bone quality possibly partly through enhancing OVX rats’ antioxidant enzymes activities and reducing bone mineral elements.

## Figures and Tables

**Table 1.** Body weight, femoral dry weight, femoral mineral content, and density of experimental animals.

**Group**	**Body weight (g)**	**Femoral dry weight (g)**	**Total femoral BMC (g)**	**Total femoral BMD (g/cm^2^)**
sham	487.2 ± 26.3	1.231 ± 0.025	0.871 ± 0.024	0.315 ± 0.007
OVX	433.1 ± 30.4 ^a^	1.101 ± 0.019 ^b^	0.794 ± 0.02 ^b^	0.279 ± 0.007 ^b^
OVX + CHE (100 mg kg^−1^ daily)	461.2 ± 24.5 ^c^	1.106 ± 0.027	0.811 ± 0.021	0.298 ± 0.006 ^c^
OVX + CHE (200 mg kg^−1^ daily)	478.3 ± 25.9 ^c^	1.119 ± 0.031 ^c^	0.866 ± 0.028 ^d^	0.311 ± 0.009 ^d^

a *p* < 0.01,

b *p* < 0.01, compared with sham control;

c *p* < 0.05,

d *p* < 0.01, compared with OVX group.

**Table 2.** Effect of *Cistanches Herba* extract treatment on bone biomechanical data.

**Group**	**Sham**	**OVX**	**OVX + CHE (100 mg kg^−1^ daily)**	**OVX + CHE (200 mg kg^−1^ daily)**
Maximum load (kN)	0.113 ± 0.018	0.096 ± 0.013 ^b^	0.107 ± 0.011 ^c^	0.111 ± 0.013 ^c^
Displacement at maximum load (mm)	0.602 ± 0.078	0.532 ± 0.027 ^b^	0.583 ± 0.032 ^c^	0.616 ± 0.031 ^d^
Stress at maximum load (MPa)	0.743 ± 0.057	0.621 ± 0.045 ^b^	0.686 ± 0.046 ^c^	0.728 ± 0.053 ^d^
Load at auto break (kN)	0.091 ± 0.006	0.075 ± 0.006 ^b^	0.081 ± 0.01	0.092 ± 0.009 ^c^
Displacement at auto break (mm)	0.712 ± 0.085	0.605 ± 0.025 ^b^	0.662 ± 0.018 ^c^	0.708 ± 0.011 ^d^
Stress at auto break (MPa)	0.704 ± 0.053	0.621 ± 0.047 ^b^	0.673 ± 0.027 ^c^	0.708 ± 0.051 ^d^

b *p* < 0.01, compared with sham control;

c *p* < 0.05,

d *p* < 0.01, compared with OVX group.

**Table 3.** Effect of *Cistanches Herba* extract treatment on blood Ca, Zn and Cu levels.

**Group**	**Ca (mmol/L)**	**Zn (μmol/L)**	**Cu (μmol/L)**
sham	2.95 ± 0.09	16.29 ± 1.11	26.17 ± 0.15
OVX	3.35 ± 0.08 ^b^	19.02 ± 1.05 ^b^	29.15 ± 0.17 ^b^
OVX + CHE (100 mg kg^−1^ daily)	3.23 ± 0.11	18.13 ± 0.09 ^c^	29.04 ± 0.21
OVX + CHE (200 mg kg^−1^ daily)	3.06 ± 0.09 ^c^	17.39 ± 1.32 ^c^	26.15 ± 0.19 ^d^

b *p* < 0.01, compared with sham control;

c *p* < 0.05,

d *p* < 0.01, compared with OVX group.

**Table 4.** Effect of *Cistanches Herba* extract treatment on blood alkaline phosphatase (ALP) activity and blood malondialchehyche (MDA) level.

**Group**	**ALP (IU/L)**	**MDA (nmol/L)**
sham	89.41 ± 4.07	7.38 ± 0.45
OVX	118.53 ± 8.21 ^b^	12.03 ± 0.72 ^b^
OVX + CHE (100 mg kg^−1^ daily)	107.21 ± 9.62	10.15 ± 0.49 ^c^
OVX + CHE (200 mg kg^−1^ daily)	90.28 ± 7.28 ^c^	8.32 ± 0.88 ^d^

b *p* < 0.01, compared with sham control;

c *p* < 0.05,

d *p* < 0.01, compared with OVX group.

**Table 5.** Effect of *Cistanches Herba* extract treatment on blood superoxide dismulase (SOD), catalase (CAT) and glutathione peroxidase (GSH-Px) activities.

**Group**	**SOD**	**CAT**	**GSH-Px**
sham	195.23 ± 11.94	34.21 ± 1.73	27.03 ± 1.94
OVX	121.64 ± 9.52 ^b^	22.16 ± 1.05 ^b^	12.13 ± 1.01 ^b^
OVX + CHE (100 mg kg^−1^ daily)	164.82 ± 12.51 ^d^	28.41 ± 1.31 ^c^	18.42 ± 1.32 ^d^
OVX + CHE (200 mg kg^−1^ daily)	184.4 ± 11.49 ^d^	33.69 ± 1.29 ^d^	24.71 ± 0.93 ^d^

b *p* < 0.01, compared with sham control;

c *p* < 0.05,

d *p* < 0.01, compared with OVX group.
